# Effects of *myo*-inositol supplementation in the diet on *myo*-inositol concentrations in the intestine, blood, eggs, and excreta of laying hens

**DOI:** 10.1016/j.psj.2024.104545

**Published:** 2024-11-10

**Authors:** Vera Sommerfeld, Anna Hanauska, Korinna Huber, Jörn Bennewitz, Amélia Camarinha-Silva, Martina Feger, Michael Föller, Michael Oster, Siriluck Ponsuksili, Sonja Schmucker, Jana Seifert, Volker Stefanski, Klaus Wimmers, Markus Rodehutscord

**Affiliations:** a Institute of Animal Science, University of Hohenheim, 70599 Stuttgart, Germany; b Department of Physiology, University of Hohenheim, 70599 Stuttgart, Germany; c Research Institute for Farm Animal Biology (FBN), 18196 Dummerstorf, Germany

**Keywords:** Egg, Laying hen, Mucosal phosphatase, *Myo*-inositol, Phosphorus

## Abstract

The objectives of this study were to investigate whether an increased dietary *myo*-inositol (**MI**) supply translates into changes in MI concentrations and endogenous mucosal phosphatase activities in the intestine of laying hens and whether different laying hen strains respond differently to MI supplementation. The diets were corn–soybean meal-based and supplemented without (**MI0**) or with 1 (**MI1**), 2 (**MI2**), or 3 (**MI3**) g MI/kg feed. Ten hens per strain (Lohmann Brown-classic (**LB**) and Lohmann LSL-classic (**LSL**)) and diet were sacrificed at the age of 30 wk following a 4-wk stay in a metabolic unit. The blood plasma, digesta of the duodenum+jejunum and distal ileum, mucosa of the duodenum, and eggs were collected at wk 30. The concentration of MI in the blood plasma was increased by MI supplementation (*P* < 0.001); however, that of MI3 did not further increase compared with MI2. The concentration of MI in the duodenum+jejunum and ileum increased steadily (*P* < 0.001). The MI concentration in the duodenum+jejunum was higher in LB than in LSL hens (*P* = 0.017). The MI concentration in egg yolk was increased by MI supplementation (*P* < 0.001) and was higher in LB than in LSL hens (*P* = 0.015). Strain or diet did not affect mucosal phosphatase activity. *Myo*-inositol flow at the terminal ileum and postileal disappearance increased with each increment in MI supplementation (*P* < 0.001) and was higher in LB than in LSL hens (*P* ≤ 0.041). Regression analysis indicated that, on average, 84% of supplemented MI was retained in the body or metabolized and excreted in a different form. Based on the measured MI concentrations in the blood and eggs, dietary MI was not completely absorbed in the small intestine and, to a different extent, in the two laying hen strains. A higher dietary MI supply was followed by higher intestinal absorption or metabolism by microorganisms. The fate of supplemented MI and its relevance to birds warrant further research.

## Introduction

*Myo*-inositol (**MI**), the most abundant form of inositol (cyclohexane-1,2,3,4,5,6-hexol), is a cyclic polyalcohol that has numerous functions and plays a significant role in poultry metabolism (Gonzalez-Uarquin et al., 2020b). *Myo*-inositol is involved in cell membranes as a component of phospholipids, cell signaling pathways, the development of nerves and the central nervous system, and glucose and insulin metabolism. Some studies have suggested an increase in the performance of broiler chickens with increased intestinal MI availability, and an increase in plasma serotonin and dopamine concentrations has been observed after MI supplementation of the feed (Gonzalez-Uarquin et al., 2020b). *Myo*-inositol can be synthesized de novo from glucose, provided in its free form, or released from dietary phytate in the digestive tract. However, the release of MI from phytate by laying hens is low when phytase is not added to the feed. Poultry have limited endogenous phosphatase activity. Owing to the high dietary Ca supply to laying hens, the Ca concentration in their gastrointestinal tract is high, which can further reduce endogenous phytate degradation (Sommerfeld et al., 2020b). Nevertheless, differences between the laying hen strains Lohmann Brown-Classic (**LB**) and Lohmann LSL-Classic (**LSL**) were found. The MI concentrations in egg fractions and blood was higher in LB than LSL hens, indicating metabolic differences between strains that were possibly caused by higher phosphatase activity in the brush border membrane (**BBM**) of LB hens (Sommerfeld et al., 2020a). Kidney *myo*-inositol oxygenase (**MIOX**), a key enzyme in MI metabolism in terms of MI degradation, was more highly expressed in LB than in LSL hens (Gonzalez-Uarquin et al., 2021). Supplementation with free MI led to increased MI concentrations in the small intestine and blood plasma in broiler chickens (Sommerfeld et al., 2018a; Pirgozliev et al., 2019). The effects of free MI in feed have rarely been studied in laying hens. However, an increase in MI concentration in the gizzard, ileum, blood, and egg yolk was observed after adding 0.16% free MI to the diets of laying hens (Herwig et al., 2021).

The endogenous mucosal phosphatase activity of poultry varies and depends on the feed composition. Phytase supplementation increased mucosal phosphatase activity in broilers and turkeys (Novotny et al., 2023a,b). This was hypothesized to be mediated by increased concentrations of Ins(1,2,5,6)P_4_ in the gizzard, which triggers phosphatases in the small intestine. However, MI concentration simultaneously increased, suggesting that MI also affected endogenous phosphatases. Furthermore, dietary supplementation with MI increased the transcript abundance of alkaline phosphatase in the ileal mucosa (Herwig et al., 2021). The authors assumed the rephosphorylation of MI when present at high concentrations, triggering phosphatases, as already suggested by Pirgozliev et al. (2019).

The objective of this study was to investigate the responses of two laying hen strains to an increased dietary MI supply, considering the MI concentrations and endogenous mucosal phosphatases in the intestine, MI in the egg, and MI excretion. It was hypothesized that increasing levels of MI provided with feed would be absorbed in the intestine and transferred to the egg to different extents in laying hen strains.

## Materials and methods

This study was part of the interdisciplinary Research Unit P-Fowl – Inositol phosphates and *myo*-inositol in the domestic fowl: Exploring the interface of genetics, physiology, microbiome, and nutrition (https://p-fowl.uni-hohenheim.de/). The animal trial was conducted at the Agricultural Experiment Station of the University of Hohenheim, Germany. It was approved by the Regierungspräsidium Tübingen, Germany (Project no. HOH67-21TE) and conducted in accordance with German Animal Welfare Legislation.

### Birds and housing

A total of 240 LB and 240 LSL newly-hatched female chickens were obtained from a breeding company (Lohmann Breeders GmbH, Cuxhaven, Germany), representing two distinct genetic backgrounds. For each strain, 12 non-related roosters were used for egg production, and 20 offspring per rooster were initially selected.

The chickens were raised in floor pens with deep litter bedding according to the standardized routine procedure of the experimental station before being moved to metabolic units at 26 wk of age. Prior to placement, offspring of 10 roosters per strain were selected based on average BW. One randomly chosen offspring from each of the 10 roosters per strain was placed in one metabolism unit (1 m × 1 m × 1 m) in a randomized complete block design 4 wk before slaughter, resulting in 10 replicates per strain and diet (*n* = 80). The metabolism units were equipped with a wooden perch, nest, feeding trough, water cups, and wire mesh floor. Metal trays were installed underneath to collect excreta. The photoperiod was 16 h of light and 8 h of darkness. The temperature in the barn was set to 18–22°C.

### Diets

The experimental diets were based on corn and soybean meal to minimize the intrinsic plant phytase activity (Table 1). Diets were calculated to contain 2.2 g non-phytate P (**nPP**)/kg based on recent suggestions (Rodehutscord et al., 2023) and were adequate for all other nutrients according to the recommendations of the Gesellschaft für Ernährungsphysiologie (**GfE**) (Gesellschaft für Ernährungsphysiologie, 1999). Diets contained TiO_2_ as an indigestible marker and were provided in a mash form. The diets were mixed in a certified feed mill at the Agricultural Experiment Station of the University of Hohenheim. The experiment was designed as a 2 × 4 factorial arrangement of treatments (2 laying hen strains and 4 MI levels) and included diets without MI supplementation (0 g/kg; **MI0**; Thermo Fisher GmbH, Kandel, Germany), 1 g MI/kg (**MI1**), 2 g MI/kg (**MI2**), or 3 g MI/kg (**MI3**). The chosen supplementation levels simulated approximately half or complete release of MI contained in the feed as InsP_6_ (1 and 2 g MI/kg), or a therapeutic dose level as applied in humans (3 g MI/kg). Feed and water were provided for ad libitum consumption. The calculated nutrient concentrations were confirmed using various analyses (Table 1).

**Table 1:** Ingredients as well as the calculated and analyzed compositions of the experimental diets.

*Ingredients, g/kg*	MI0	MI1	MI2	MI3
Corn	591	590	589	588
Extracted soybean meal	260	260	260	260
Alfalfa meal	30	30	30	30
Soybean oil	20	20	20	20
DL-Methionine	3.5	3.5	3.5	3.5
Monocalcium phosphate	4.7	4.7	4.7	4.7
Limestone, fine	23	23	23	23
Limestone, coarse	54.4	54.4	54.4	54.4
Sodium chloride	3	3	3	3
Choline chloride	1	1	1	1
Sodium bicarbonate	1.9	1.9	1.9	1.9
Vitamin mix^1^	2	2	2	2
Mineral mix^2^	0.5	0.5	0.5	0.5
*myo*-inositol^3^	0	1	2	3
TiO_2_	5	5	5	5
*Calculated, g/kg*				
*myo*-inositol	0.4	1.4	2.4	3.4
P	4.0	4.0	4.0	4.0
Non phytate-P	2.2	2.2	2.2	2.2
Ca	35.0	35.0	35.0	35.0
CP	168	168	168	168
ME, MJ/kg	11.6	11.6	11.6	11.6
*Analyzed, g/kg DM*				
*myo*-inositol	0.37	1.51	2.76	3.72
P	4.4	4.5	4.4	4.5
Ca	34.6	36.3	33.3	33.3

1 Vitamin premix (Miavit GmbH, Essen, Germany), provided per kg of the complete diet: 10,000 IU vitamin A, 3,000 IU vitamin D3, 30 mg vitamin E, 2.4 mg vitamin K3, 100 mcg biotin, 1 mg folic acid, 3 mg vitamin B1, 6 mg vitamin B2, 6 mg vitamin B6, 30 mcg vitamin B12, 50 mg nicotinamide, 14 mg calcium-D-pantothenate.

2 Trace element premix (Gelamin Gesellschaft für Tierernährung mbH, Memmingen, Germany), provided per kg of complete diet: 80 mg manganese from manganese-(II)oxide, 60 mg zinc from zinc sulfate monohydrate, 25 mg iron from ferrous-(II)-sulfate monohydrate, 7.5 mg copper from cupric-(II)-sulfate pentahydrate, 0.6 mg iodine from calcium iodate, 0.2 mg selenium from sodium selenite.

3 Thermo Fisher GmbH, Kandel, Germany.

### Experimental procedures, samplings and measurements

The experimental diets were provided to the hens for 4 wk starting with placement in the metabolism units at wk 26. Animals were inspected at least twice daily. The eggs were collected and weighed. One egg per hen was taken during the 4 excreta collection days and kept at room temperature until further processing for MI analysis. Feed and individual hens were weighed at the beginning and end of the 4 wk and at the beginning and end of the excreta collection days. Total excreta were collected from the trays at 24-h intervals for 4 consecutive days in the week prior to slaughter. Feathers, skin scales, and spilled feed were carefully removed prior to excreta collection. Feed residues from the trays were weighed and feed intake was corrected for these residues. Excreta were immediately frozen after each collection at −20°C.

Before slaughtering 20 hens per day in wk 30, feed was deprived for 1 h, followed by 1 h ad libitum access to feed to standardize the gut fill. The hens were individually stunned with a gas mixture of 35% CO_2_, 35% N_2_, and 30% O_2_ and sacrificed by decapitation. Trunk blood was collected in tubes containing sodium fluoride for MI analysis or lithium heparin for P and Ca analyses. Blood samples were centrifuged for 10 min at 2,500 × *g* to obtain plasma. Digesta from the duodenum and jejunum together (duodenum+jejunum) and the terminal part of the ileum, defined as the last two-thirds of the section between the Meckel´s diverticulum and 2 cm prior to the ileo-ceco-colonic junction, were collected by gentle squeezing. Digesta samples were immediately frozen at −20°C, freeze-dried, and pulverized (PULVERISETTE 9, Fritsch GmbH, Idar-Oberstein, Germany). The pulverized samples were stored in airtight containers until further analysis.

After removal of the digesta, the duodenum was opened longitudinally and cleaned in cold physiological saline solution (0.9% NaCl). The mucosa from the whole section was stripped off on an ice bed with two microscopic slides, shock-frozen in liquid nitrogen, transported in liquid nitrogen to the laboratory, and stored at −80°C until further analysis.

The yolk and albumen from one egg per hen were weighed separately, frozen, freeze-dried, weighed again, pulverized using a mortar and pestle, and stored until further analysis.

### Sample preparation and analyses

Excreta samples were thawed at 3°C, weighed, pooled for each hen, and homogenized. Excreta DM was analyzed in triplicate. A subsample of the excreta was freeze-dried and pulverized. The pulverized samples were stored in airtight containers until further analysis. The feed was ground to pass through a 0.5 mm sieve (Ultra Centrifugal Mill ZM 200, Retsch GmbH, Haan, Germany). Excreta samples were analyzed for DM according to the official method in Germany (method no. 3.1) (Verband Deutscher Landwirtschaftlicher Untersuchungs- und Forschungsanstalten (VDLUFA), 2007).

Pulverized feed, digesta, yolk, albumen, plasma, and excreta samples were analyzed for MI according to Sommerfeld et al. (2018b) using gas chromatography/mass spectrometry after derivatization. Calcium and inorganic P (**P_i_**) in the blood plasma were analyzed by IDEXX BioResearch Vet Med Labor GmbH (Ludwigsburg, Germany). Calcium was measured photometrically using the Arsenazo method and P_i_ as phosphomolybdate complex using a Beckman Olympus AU480 instrument.

Brush border membranes were enriched, and mucosal phosphatase levels were determined, as previously described by Huber et al. (2015) and Gonzalez-Uarquin et al. (2020a), with slight modifications. Briefly, BBM was ground in liquid nitrogen and homogenized before enrichment by MgCl_2_ precipitation of the basolateral membranes. The phosphatase activity was determined with the K-PHYT test kit (Megazyme International, Ireland) using an amount of BBM equivalent to 160 µg BBM protein and 25 µg sodium phytate. The assay was run at pH 5.5 and 40°C for 15 min. Free P_i_ was measured photometrically at 655 nm. The received values were converted to µmol P_i_/g BBM protein/min.

### Calculations and statistical analysis

According to the common technical terms used in balance studies, the difference between MI intake and MI excretion (feces and urine) was calculated as MI retention:

(1) $$\begin{array}{c} \mathrm{MI}\;\mathrm{retention}\;\left(\%\right)=\left(\mathrm{MI}\;\mathrm{intake}\;\left(\mu \mathrm{mol}/d\right)\;-\;\mathrm{MI}\;\mathrm{in}\;\mathrm{excreta}\;\left(\mu \mathrm{mol}/d\right)\right)\;/\;\mathrm{MI}\;\mathrm{intake}\;\left(\mu \mathrm{mol}/d\right)\;\times \;100 \end{array}$$

Notably, this does not include the urinary excretion of compounds originating from MI metabolism in animals. However, urinary excretion of MI is thought to be very low in non-diabetic animals because of the high reabsorption of MI from the kidney into the blood (Daughaday et al., 1954).

Alternatively, the MI retention was calculated by subtracting the MI in the egg from the MI intake.

(2) $$\begin{array}{c} \mathrm{MI}\;\mathrm{retention}\;\left(\%\right)\;=\;\left(\mathrm{MI}\;\mathrm{intake}\;\left(\mu \mathrm{mol}/d\right)\;-\;\mathrm{MI}\;\mathrm{in}\;\mathrm{excreta}\;\left(\mu \mathrm{mol}/d\right)\;-\;\mathrm{MI}\;\mathrm{in}\;\mathrm{eggs}\;\left(\mu \mathrm{mol}/d\right)\right)\;/\;\mathrm{MI}\;\mathrm{intake}\;\left(\mu \mathrm{mol}/d\right)\;\times \;100 \end{array}$$

The MI flow at the terminal ileum was calculated as

(3) $$\begin{array}{c} \mathrm{MI}\;\mathrm{flow}\;\left(\mu \mathrm{mol}/d\right)\;=\;\mathrm{MI}\;\mathrm{inta}\mathrm{ke}\;\left(\mu \mathrm{mol}/d\right)\;\times \;\left(100\;-\;\mathrm{prececal}\;\mathrm{MI}\;\mathrm{disappearance}\;\left(\%\right)\right)\;/\;100 \end{array}$$

The postileal MI disappearance was calculated as

(4) $$\begin{array}{c} \mathrm{Postileal}\;\mathrm{MI}\;\mathrm{disappearance}\;\left(\mu \mathrm{mol}/d\right)\;=\;\mathrm{MI}\;\mathrm{flow}\;\mathrm{at}\;\mathrm{the}\;\mathrm{terminal}\;\mathrm{ileum}\;\left(\mu \mathrm{mol}/d\right)\;-\;\mathrm{MI}\;\mathrm{excretion}\;\left(\mu \mathrm{mol}/d\right) \end{array}$$

Statistical comparisons were performed using the MIXED procedure and pairwise t-tests using the SAS software package (version 9.3; SAS Institute Inc., Cary, North Carolina, USA). Individual hens were considered as the experimental unit. The following model was used:

$$Y_{\text{ijkl}}=\mu +\alpha_{i}+\beta_{j}+{\left(\alpha \beta \right)}_{\text{ij}}+\gamma_{k}+\phi_{l}+\varepsilon_{\text{ijkl}},$$

where Y_ijkl_ = response variable, μ = overall mean, α_i_ = effect of strain (fixed), β_j_ = effect of MI supplementation (fixed), the interaction between strain and MI supplementation (fixed), γ_k_ = block (random), ϕ_l_ = father/rooster (random), and ε_ijkl_ = residual error. Polynomial orthogonal contrasts were constructed to test the linear and quadratic effects of MI supplementation when the main effect was significant. Statistical significance was set at *P* < 0.05, with trends at *P* < 0.10.

## Results

The concentration of MI in the blood plasma was increased linearly and quadratically by MI supplementation (*P* < 0.001); however, the MI2 and MI3 values did not differ (Table 2). The MI concentration in the duodenum+jejunum was increased linearly and in the ileum linearly and quadratically with each increase in MI supplementation (*P* < 0.001). The MI concentration in the duodenum+jejunum was higher in LB than in LSL hens (*P* = 0.017). The MI concentration in egg yolk was linearly increased by MI supplementation (*P* < 0.001) and was higher in LB than in LSL hens (*P* = 0.015). Strain or diet did not affect mucosal phosphatase activity (*P* > 0.05).

**Table 2:** Effects of different dietary *myo*-inositol (MI) concentrations on MI concentrations in plasma, small intestine, and egg fractions, and phosphatase activity in the duodenum of two laying hen strains at 30 wk of age.

		Plasma MI	Duodenum+Jejunum MI	Ileum MI	Albumen MI	Yolk MI	Phosphatase Activity
Strain	Diet	mmol/l	µmol/g DM	µmol/g DM	µmol/g DM	µmol/g DM	µmol P_i_/g BBM protein/min
LB^1^	MI0^3^	0.15	7.8	2.6	3.9	1.9	5.5
LB	MI1^4^	0.20	15.0	7.4	4.1	2.0	5.9
LB	MI2^5^	0.24	26.5	19.0	4.5	2.0	6.4
LB	MI3^6^	0.26	33.9	31.7	4.3	2.2	5.9
LSL^2^	MI0	0.13	8.1	2.4	4.0	1.7	5.1
LSL	MI1	0.20	14.9	7.6	4.2	1.8	4.9
LSL	MI2	0.24	20.8	14.1	4.2	1.9	5.1
LSL	MI3	0.23	27.6	24.1	4.5	2.1	5.7
pooled SEM		0.011	1.53	2.01	0.28	0.06	0.64
LB			20.8^a^			2.0^a^	
LSL			17.8^b^			1.9^b^	
	MI0	0.14^c^	8.0^d^	2.5^d^		1.8^c^	
	MI1	0.20^b^	14.9^c^	7.5^c^		1.9^b^	
	MI2	0.24^a^	23.7^b^	16.5^b^		2.0^b^	
	MI3	0.25^a^	30.8^a^	27.9^a^		2.1^a^	
*P* values							
Strain		0.101	0.017	0.078	0.909	0.015	0.163
Diet		< 0.001	< 0.001	< 0.001	0.093	< 0.001	0.745
Strain × Diet		0.217	0.053	0.120	0.471	0.680	0.711
Linear effect of diet		< 0.001	< 0.001	< 0.001		< 0.001	
Quadratic effect of diet		< 0.001	0.941	0.021		0.784	

Data are given as LSmeans; *n* = 10 hens.

1 LB = Lohmann Brown-Classic.

2 LSL = Lohmann LSL-Classic.

3 without MI supplementation.

4 1 g supplemented MI/kg.

5 2 g supplemented MI/kg.

6 3 g supplemented MI/kg.

*Myo*-inositol intake, MI flow at the terminal ileum, and postileal MI disappearance increased linearly, and MI intake and MI flow also quadratically with each increase in MI supplementation (Table 3; *P* < 0.001), and the values were higher in LB than in LSL hens (*P* ≤ 0.041). The concentration of MI in the excreta and MI excretion increased linearly and quadratically with each MI supplementation level (*P* < 0.001). Between 83 and 86% of MI intake was not recovered in excreta and eggs when MI was supplemented, and 69% when MI was not supplemented.

**Table 3:** Effects of different dietary *myo*-inositol (MI) concentrations on MI intake, ileal flow, excretion, and retention of two laying hen strains at 30 wk of age.

		MI intake	MI flow ileum^1^	Postileal MI disappearance^2^		MI concentration in excreta		MI excretion	MI retention^3^	MI retention subtracting eggs^4^
Strain	Diet	µmol/d	µmol/d	µmol/d		µmol/g DM		µmol/d	%	%
LB^5^	MI0^7^	211	86	52		1.2		34	84	71
LB	MI1^8^	872	289	190		3.1		99	89	86
LB	MI2^9^	1715	737	504		7.2		233	86	85
LB	MI3^10^	2223	1177	801		11.8		376	83	82
LSL^6^	MI0	216	87	46		1.4		41	81	67
LSL	MI1	883	241	150		3.0		91	90	86
LSL	MI2	1597	531	336		6.6		195	88	86
LSL	MI3	2139	879	581		10.1		298	86	84
pooled SEM		32.5	62.2	60.7		0.62		21.0	1.5	1.6
LB		1255^a^	572^a^	387^a^						
LSL		1209^b^	434^b^	278^b^						
	MI0	214^d^	86^d^	49^d^		1.28^d^		38^d^	82^c^	69^b^
	MI1	878^c^	265^c^	170^c^		3.06^c^		95^c^	89^a^	86^a^
	MI2	1656^b^	634^b^	540^b^		6.90^b^		214^b^	87^ab^	85^a^
	MI3	2181^a^	1028^a^	691^a^		10.94^a^		337^a^	85^bc^	83^a^
*P* values										
Strain		0.048	0.012	0.041		0.297		0.072	0.644	0.889
Diet		<0.001	<0.001	<0.001		<0.001		<0.001	<0.001	<0.001
Strain × Diet		0.125	0.054	0.192	0.391		0.199		0.194	0.130
Linear effect of diet		< 0.001	< 0.001	< 0.001	< 0.001		< 0.001		0.320	< 0.001
Quadratic effect of diet		0.004	0.014	0.067	0.010		0.030		< 0.001	< 0.001

Data are given as LSmeans; *n* = 10 hens.

1 equation (3)

2 equation (4)

3 equation (1)

4 equation (2)

5 LB = Lohmann Brown-Classic.

6 LSL = Lohmann LSL-Classic.

7 without MI supplementation.

8 1 g supplemented MI/kg.

9 2 g supplemented MI/kg.

10 3 g supplemented MI/kg.

Mean BW and egg weight were higher in LB than in LSL hens (Table 4; *P* < 0.001 and *P* = 0.031, respectively). The ADFI during the excreta sampling period and blood plasma concentrations of P_i_ and Ca were not affected by the strain or diet (*P* > 0.05).

**Table 4:** Effects of different dietary *myo*-inositol (MI) concentrations on performance and plasma traits of two laying hen strains at 30 wk of age.

		BW^1^	ADFI^2^	Egg weight^2^	Plasma P	Plasma Ca
Strain	Diet	g	g/d	g	mmol/L	mmol/L
LB^3^	MI0^5^	1889	112	63	1.9	7.8
LB	MI1^6^	1890	116	63	1.8	7.8
LB	MI2^7^	1914	125	63	1.8	7.8
LB	MI3^8^	1977	120	66	2.0	8.3
LSL^4^	MI0	1640	115	60	1.8	7.6
LSL	MI1	1624	117	62	1.8	7.8
LSL	MI2	1631	116	62	1.8	7.5
LSL	MI3	1602	116	61	2.0	8.4
pooled SEM		31.3	3.2	1.2	0.10	0.41
LB		1917^a^		64^a^		
LSL		1624^b^		61^b^		
*P* values						
Strain		<0.001	0.330	0.031	0.707	0.697
Diet		0.634	0.206	0.403	0.143	0.287
Strain × Diet		0.088	0.274	0.416	0.880	0.971

Data are given as LSmeans; *n* = 10 hens.

1 end of excreta sampling.

2 during 4-d sampling period.

3 LB = Lohmann Brown-Classic.

4 LSL = Lohmann LSL-Classic.

5 without MI supplementation.

6 1 g supplemented MI/kg.

7 2 g supplemented MI/kg.

8 3 g supplemented MI/kg.

## Discussion

*Myo*-inositol fulfills many functions in animal metabolism, and several effects of MI, either released from phytate or offered in free form in poultry, have been considered (Lee and Bedford, 2016; Gonzalez-Uarquin et al., 2020b). Differences were found in MI concentrations in the blood and egg fractions and kidney MIOX expression between LB and LSL hens fed diets without supplemented phytase or MI (Sommerfeld et al., 2020a; Gonzalez-Uarquin et al., 2021). These observations, based on differences in the endogenous potential of the hens to synthesize and metabolize MI, were the motivation for the present study in which different MI supplementation levels were offered to the two laying hen strains.

### Myo*-inositol absorption*

The MI concentrations in the duodenum+jejunum, ileum, and egg yolk were either significantly higher or tended to be higher in LB than in LSL hens, which partly confirmed the results of Sommerfeld et al. (2020a). This might indicate morphological differences in the length of the intestine or a different absorption capacity for MI between the strains. The concentration of MI in the duodenum+jejunum and ileum in both strains increased dose-dependently. This is plausible as more MI reached the intestine with every supplementation level. In contrast, increased concentrations indicated that MI was not entirely absorbed by the tissues or used by microorganisms during passage through the small intestine. *Myo*-inositol is believed to be completely absorbed in the small intestines of different species (Croze and Soulage, 2013). However, the results of the studies that are cited in this context were based on the minimal recovery of MI in the feces of humans (0.2% of intake) (Clements and Reynertson, 1977) and rats (4% of intake) (Nahapetian and Young, 1980). Although these authors did not exclude the possibility of MI being partially used by microorganisms or converted to intermediate metabolites, the misconception of the complete absorption of MI remains to date. However, several studies indicated only partial intestinal absorption of MI. As early as 1954, Daughaday et al. (1954) observed an intestinal MI absorption of 26% in rats 6 h after MI ingestion, with an average absorption of 3.4 mg MI/100 g BW/hour. Large differences were found among individuals when intestinal segments were incubated with MI, and MI uptake into the tissue was measured, with values ranging from 2 to 113 nmol/g of wet intestine/10 min (Lerner and Smagula, 1979). Several studies have reported increasing MI concentrations in the small intestine with increasing dietary MI or phytase supplementation in broilers and laying hens (Sommerfeld et al., 2018a; Herwig et al., 2021; Novotny et al., 2023a,b), indicating incomplete MI absorption. Similarly, in pigs, MI concentrations in the ileal digesta increased when phytase was supplemented; however, fecal MI excretion was negligible (Rosenfelder-Kuon et al., 2020; Klein et al., 2023). Walk et al. (2018) suggested, based on their observation of high ileal MI concentrations after supplementation with increasing doses of phytase in the terminal ileum, a rate-limiting step in MI absorption, possibly due to increased Na digestibility with a reduction in the H^+^ ions needed for MI transport. However, this suggestion may only apply to phytase-supplemented treatments. Klein (2024) estimated a prececal MI disappearance of approximately 10–20% in pigs fed diets without supplemented phytase and 20–40% in pigs fed diets with supplemented phytase, where very little MI was found in the feces in both variants. This exemplifies the significant discrepancy between prececal and fecal disappearance and indicates the marked involvement of hindgut microorganisms or postileal absorption. In the present study, the ileal MI flow suggested a range of MI absorption between 45 and 75% when calculated using the concentrations of free MI. Furthermore, the different MI concentrations in the blood plasma in the present study confirmed that some of the MI in the intestine was absorbed. This is consistent with the results of several previous studies on broiler chickens (Sommerfeld et al., 2018a,b; Novotny et al., 2023a,b) and pigs (Rosenfelder-Kuon et al., 2020; Klein et al., 2023). However, the stepwise increase in the small intestinal MI concentration was not found to the same extent in the blood plasma and egg yolk. The uptake of MI from the blood pool into organs and tissues is likely faster than the pool supplied by absorbed MI.

### Myo*-inositol in the eggs*

**Fig. 1:**
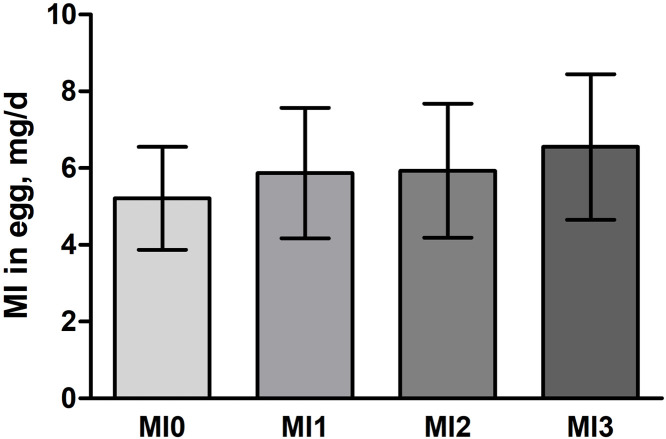
Mean amount of *myo*-inositol (MI) ± SD in eggs of two laying hen strains in dependence on different dietary MI supplementation levels (MI0 = without MI supplementation, MI1 = 1 g supplemented MI/kg, MI2 = 2 g supplemented MI/kg, MI3 = 3 g supplemented MI/kg).

Although the MI content in the eggs significantly increased with MI supplementation, the difference between the unsupplemented and supplemented diets was not large overall (Fig. 1). This indicated that the most relevant proportion of MI in eggs stems from an unknown mixture of MI released from phytate, free MI contained in the common feed ingredients, and de novo synthesis. As the amount of free MI in the feed was low and phytate degradation is only moderate in laying hens (van der Klis et al., 1997; Siegert et al., 2023), de novo synthesis of MI seems to be the most plausible cause and, therefore, relevant for MI transfer to the egg, perhaps even more relevant than the very high amounts of free MI supplemented in the diets. However, it cannot be ruled out that a proportion of the MI was implemented in other metabolites such as phospholipids (Lee and Bedford, 2016). Measurements of MI-containing components or MI-converting enzymes might clarify this issue.

### Myo*-inositol retention*

**Fig. 2:**
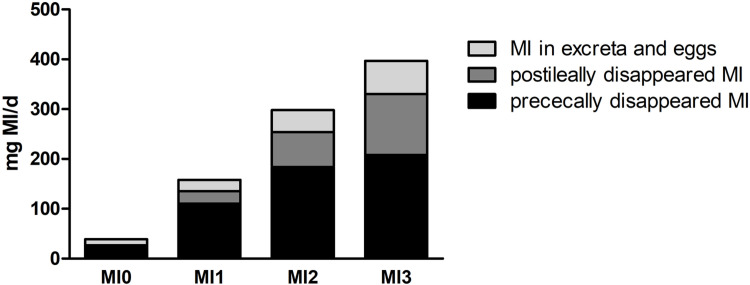
Calculated amount of prececal and postileal *myo*-inositol (MI) disappearance and amount in excreta and eggs of two laying hen strains in dependence of different dietary MI supplementation levels (MI0 = without MI supplementation, MI1 = 1 g supplemented MI/kg, MI2 = 2 g supplemented MI/kg, MI3 = 3 g supplemented MI/kg). The complete bar represents the daily MI intake.

With increased MI ingestion, more disappeared prececally and postileally and more left the hen via the excreta and eggs (Fig. 2). When more MI is supplied, more MI is either absorbed and transported to organs and tissues or metabolized by microorganisms in the small intestine. Furthermore, a high amount of MI appeared to disappear postileally. Hu et al. (2022) found an Na/*myo*-inositol cotransporter (SMIT) expressed at the mRNA level in the colon of pigs, which was decreased by high dietary Ca. This indicated possible MI absorption in the hindgut. However, whether this transporter is relevant to absorption needs to be verified at the protein level. Despite the possibility of MI absorption in the hindgut, it is more plausible that microorganisms in the ceca and colon metabolize MI, as some microorganisms use MI as an energy source (Weber and Fuchs, 2022).

**Fig. 3:**
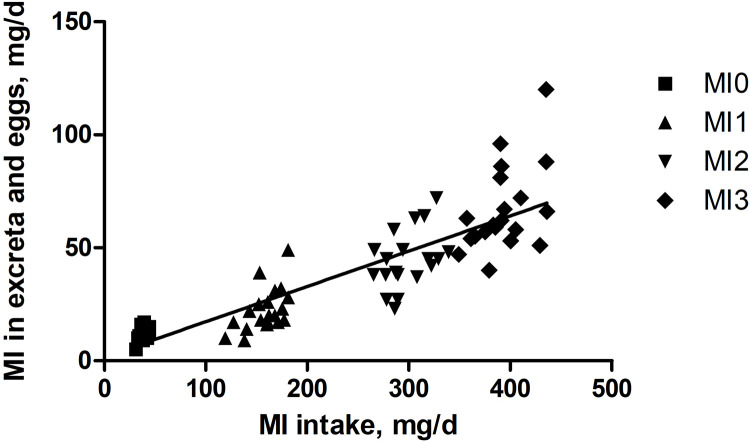
Relationship between *myo*-inositol (MI) intake and MI in excreta and eggs during the excreta sampling period of two laying hen strains. Y = 0.16 x + 1.78, *R*^2^ = 0.76. The symbols indicate data points of the individual hens.

When plotting MI intake against the MI that was not recovered in the excreta and eggs, the regression slope indicated that 0.16 mg MI/1 mg MI intake was deposited by the animal (Fig. 3). The reverse means that 84% of the ingested MI either remained in the animal (including microbiota) or was removed in a different form than free MI. Regarding the latter option, MI is mainly catabolized to glucuronic acid in the kidneys via the enzyme MIOX. Glucuronic acid can then be further converted to D-xylulose-5-phosphate, which can enter the pentose phosphate pathway (Gonzalez-Uarquin et al., 2020b) and be transformed into glucose, CO_2_, and other metabolites.

### Myo*-inositol and mucosal phosphatases*

Dietary supplementation with MI increased the transcript abundance of alkaline phosphatase in the ileal mucosa (Herwig et al., 2021) and blood (Pirgozliev et al., 2019). Furthermore, including phytase in the diet not only increased the concentration of less phosphorylated InsP and MI in the digestive tract but also jejunal mucosal phosphatase activity in broilers and turkeys (Novotny et al. 2023a,b). Therefore, we hypothesized that MI might affect mucosal phosphatase activity. However, the mucosal phosphatase activity was not affected by MI supplementation in the present study. Surprisingly, we did not observe a significant effect of the laying hen strain on phosphatase activity, which is in contrast to Sommerfeld et al. (2020a). However, in Sommerfeld et al. (2020a), the activity was determined in the jejunal mucosa but not in the duodenal mucosa, as in the present study. Although InsP degradation was not measured, results on mucosal phosphatase activity and P_i_ and Ca in blood plasma indicated that MI did not interfere with phytate or P metabolism in the intestine.

### Myo*-inositol and hen performance*

MI supplementation did not affect the performance traits. This is in contrast to the findings of several studies investigating the effects of MI on poultry. However, Zyla et al. (2012) also reported no, or even negative, effects of MI on laying hen performance. It is possible that the effects of MI on performance (Zyla et al., 2004, 2013; Pirgozliev et al., 2017; Sommerfeld et al., 2018a) are visible during the early development of fast-growing poultry. The metabolic focus in laying hens is on egg production instead of growth, with a pronounced reallocation of micro- and macronutrients.

### Conclusion

Based on the MI responses found in the blood and eggs, dietary MI is absorbed in the small intestine, but not completely, and to different extents in the two laying hen strains. A higher dietary MI supply leads to higher intestinal absorption or catabolism by intestinal microorganisms. The fate of this additional MI and its metabolic relevance to animals and microbes warrants further research.

## Declaration of competing interest

MFö received speaker fees unrelated to this study from Kyowa Kirin. The authors declare no conflicts of interest.
